# P008: Salvaging catheters in the era of extensive gram-negative resistance

**DOI:** 10.1186/2047-2994-2-S1-P8

**Published:** 2013-06-20

**Authors:** N Gupta, R Soman, J Kothari, A Almeida, A Shetty, C Rodrigues

**Affiliations:** 1 Deparment of Internal Medicine & Infectious Diseases, P.D.Hinduja National Hospital & MRC , Mumbai, India; 2 Department of Nephrolgy, P.D.Hinduja National Hospital & MRC , Mumbai, India; 3 Department of Microbiology, P.D.Hinduja National Hospital & MRC , Mumbai, India

## Introduction

Central-line associated blood stream infection (CLABSIs)is associated with increase hospital costs and length of stay. Antibiotic lock therapy (ALT) along with systemic antibiotics appears to be an option for catheter salvage. Most studies on ALT have focused on Coagulase-negative staphylococcal (CoNS) infections. However, CLABSIs due to extended spectrum β- lactamase (ESBL)-producing Gram-negative bacteria (GNB) is more common in our setting.

## Objectives

Carbapenems are unsuitable for use in ALT because of instability at body temperature. Hence, there is a need to explore the use of other antimicrobials like tigecycline and colistin for antibiotic-lock solutions for management of CLABSIs due to GNB.

## Methods

Patients received ALT if they had CLABSI or were symptomatic with a colonized catheter. The antibiotic-lock solution was instilled into the retained catheter lumen for dwell times of 24 hours, and therapy was continued for 14 days. For ALT for Gram-negative CLABSIs we used colistin, tigecycline, ciprofloxacin, gentamicin and amikacin. An in vitro stability test of colistin was done with heparin and 20 % N-acetylcystiene (NAC) by bioassay using a disc diffusion method and *Bordotella bronchoseptica* ATCC 4617.

## Results

There were 10 patients with long-term intravascular devices who developed 12 episodes of bacteremia. Among the 12 episodes, 6 episodes were of CLABSIs and in the other 6 episodes patients had symptoms and a colonized catheter.

11 of the 12 episodes were caused by GNB and only 1 episode was caused by a Gram-positive organism, *Enterococcus*. Of the other 12 episodes, *Acinetobacter baumannii* were isolated in 3 episodes and *E. coli*, *Flavobacterium*, and *P. aeruginosa* were isolated in 2 each. The other organisms isolated were *K. pneumoniae*, and *B. cepacia* (1 episode each).

Successful treatment with ALT was observed in all of the 12 episodes. The median duration of catheter salvage was 60 days.

## Conclusion

The results suggest that these novel antibiotic lock combinations may be useful options for salvaging the catheters. This approach will reduce morbidity and the healthcare costs. Such strategies should be further evaluated for the treatment of CLABSIs in the era of Gram-negative resistance.

## Disclosure of interest

None declared

